# Treatment of Pulmonary Phthisis

**Published:** 1893-08-12

**Authors:** 


### The Hospital Clinic.

[*The Editor will be glad to receive offers of co-operation and contributions from members of the profession. All letters should be addressed to* The Editor, The Lodge, Porchester Square, London, W.]

### CITY OF LONDON HOSPITAL FOR DISEASES OF THE CHEST, VICTORIA PARK.

### Treatment of Pulmonary Phthisis.

### II.—CHRONIC PHTHISIS.

Under this heading will be taken those cases in which physical signs are more or less stationary, and pyrexia moderate or absent. Many of such cases might be more accurately described as sub-acute.

The chief part of the treatment consists in ordering a suitable and easily assimilable diet, and it is impossible to lay down an exact scale suitable for all patients. For those, however, who have a good appetite, and are free from any digestive derangement, the ordinary diet is as follows:—

Breakfast, 6 a.m.—Tea, coffee, or cocoa, milk, bread and butter, one egg.

Lunch, 9 a.m.—Milk, bread and butter.

Dinner, 12 30 p.m.—Varies between roast and boiled mutton, roast beef, stewed rabbit, and Irish stew. Rice, bread, sago, or tapioca pudding.

Tea, 5 p.m.—Cocoa, tea, or milk, bread and butter.

Supper, 7 30 p.m.—Milk or beef tea, bread and butter.

The above scale is subject to various modifications, for phthisical patients are notoriously capricious about their food, and special indulgence is shown them in this respect. Fish, chop, or chicken are in turn substituted for meat, and bacon is given for breakfast if desired, with extra quantities of milk, beef tea, and eggs. In the absence of dyspepsia the more fatty food the patient can take the better. Milk, lemonade, or beef tea are ordered for night consumption, and in many cases a glass of milk, with or without a little rum, is given on waking in the early morning. Alcohol is not given as a routine practice, but ale or stout, half a pint, is generally allowed with dinner. The best guide for giving stimulants is the patient's appetite, as if it is increased thereby they may be given safely. Of course if there is much weakness or prostration wine, whiskey, or brandy are given freely. Speaking generally, alcohol is contra-indicated in early acute, useful in chronic and advanced stages.

Of the various substances used as adjuvants to the food, cod liver oil is the one chiefly used here, and is given as a routine practice to those who can take and digest it. The dose varies from ʒi to ʒiv, taken twice or three times a day soon after meals, and beginning with the smaller quantity. When it has a nauseating effect it is generally sufficient to add a few drops of tincture of orange or an ounce of peppermint water, and another useful method of checking nausea is to suck a slice of lemon before taking it. One of the various patented emulsions is sometimes tried when the pure oil cannot be taken, but some patients are never able to overcome their dislike to cod liver oil in any form, and to these maltine is generally given, ʒi to ʒii three times a day. The use of oil, maltine, and other similar substances is contra-indicated in the presence of dyspepsia or hæmoptysis, and in acute stages.

With regard to drugs, the most important consideration is the condition of the stomach, for it is worse than useless to give full diet or cod liver oil to those who are suffering from dyspepsia. The following conditions are those most frequently met with in this disease and most important from the point of view of treatment.

An atonic condition of the stomach is very frequent, indicated by a sense of weight, or sometimes actual pain, in the epigastrium or between the shoulders, and generally increased after the ingestion of food. Nausea, flatulence, and constipation are frequently present, but the chief diagnostic sign is the large, flabby tongue, indented by the teeth at its edges, and perhaps covered with thin white fur. Tea, potatoes, beer, and all obviously indigestible articles of food are strictly forbidden, and either fish, chicken, or mutton broth substituted for meat. Two or three ounces of claret or freely diluted whiskey are given at meals if the appetite is thereby increased, and lime water or soda water given with the milk; but the most suitable diet for any one patient is often ascertained only by experiment. This condition is generally relieved by acids, combined with a muscular tonic, a favourite prescription here consisting of tinct. nucis. vom., ♏v. to x.; spirit chlorof., ♏xv.; acid nitro-hydrochor. dil., ♏xv.; inf. gent. co. ad. ℥i., given before or after each meal. Sometimes more benefit is obtained by the use of alkalies, *e g*, sodii bicarb., gr. x.; inf. gent. co. ad. ℥i., with or without a few drops of tinct. nucis vom. Pepsin is often very useful, and may be given in a dose of five grains, with acid hydrochlor., dil., ♏x.; aq. ad. ℥i., a quarter of an hour before or after meals, as experiment may decide. Papain is much used here in this condition, generally prescribed in a dose of two grains in powder to be taken before meals, but it may also be combined with either acid or alkaline mixtures. For the relief of flatulence, the addition to the medicine of sodii sulpho. carb. gr. x., or tinct. zingib. fort. ♏v., is generally sufficient. Another favourite prescription here for flatulent dyspepsia consists of sodii bicarb. gr. x., tinct. gent. co. ʒ½., spir. chlorof. ♏x, inf. rhei, aq. menth. pip. aa. ℥½., to be taken three times a day after meals. One or two minims of oil of cajuput on a lump of sugar will generally relieve the sense of oppression after food, but failing this and the above remedies, a blister over the epigastrium and a few drops of liq. morph. hyd. may be tried.

Gastric catarrh may present symptoms very similar to those of atony, but as a rule pain is more severe, nausea and vomiting more frequent, and the tongue covered with thick or dried fur, and showing signs of inflammation about the tip and edges. In the severer forms of this condition, when the patient is unable to retain even liquid food, nutrient enemata or suppositories are chiefly relied on, and iced water only given by the mouth. A nutrient enema may consist of milk and beef tea aa. ℥i., brandy ℥½, liquor pancreaticus ʒi., to which tinct. opii. ♏x. may be added if there is any difficulty in its retention, and this may be given four or five times daily. In less severe cases pain and vomiting are generally relieved by limiting the diet to fluid food, such as milk, soda water, and ice, together with a gastric sedative, *e.g.*, bismuth subnit, soda bicarb. aa. gr. x., inf. calumba ad. ℥i., to which liq. morph. hyd. ♏x., or acid hydrocyan. dil. ♏iii. may be added if necessary. Another efficacious remedy for vomiting consists of three or four drops of acid hydrocyan. dil. added to an effervescing mixture prepared with sod. bicarb. gr. xx, acid. tart. gr. xvii. As this condition improves the diet is increased cautiously, strong beef tea and pounded meat being given until fish or chicken can be taken without discomfort. The above acid or alkaline mixtures, with pepsin or papain, may be prescribed as indicated, or the following, which is largely used here, radicis rhei. contusa gr. xxv., radicis zingib gr. xv., radicis gentiani ʒi gs. sod. bicarb. ʒii., aqua frigida Oi. Prepared by macerating for twenty-four hours, and kept constantly fresh, ℥i. to be taken three times daily before meals.

Constipation may be relieved by occasional doses of compound liquorice powder, or of a mixture containing mag. sulph. gr. xx., spir. amm. aromat ʒ ss., infus. sennæ ad. ℥ij. Constipation is very frequent in phthisical patients, independently of dyspepsia, and except in very advanced cases or those in whom there is a known tendency to diarrhœa, it is usual here to ensure a daily evacuation of the bowels by means of purgatives. If it is habitual, and especially if due to atonicity of the bowels, the following pill given every night is usually successful: Ext. nucis vom. gr. $\frac{1}{4}$, ext. aloes gr. i., pulv. myrrhæ gr. ii., saponis q. s. The stronger purgatives are avoided unless absolutely necessary.

In the absence of digestive complications one of the various tonics is generally given. In cases with marked muscular debility and anorexia, strychnine is often beneficial, and is generally given here in the following mixture: Acid phosph. dil. ♏xv., liq. strych. hyd. ♏iii., spirit cholorof. ♏xv., inf. quassiæ ad ℥i three times a day after meals. Quinine also seems to increase the appetite in some cases, and is generally given in combination with strychnine or iron, for instance, quin sulph. gr. ii., liq. strych. ♏v., acid sulph. dil. ♏v,, inf. quassiæ ad. ℥i., or if iron is indicated ferri sulph. gr. i. to iii. may be substituted for the strychnine. Iron is chiefly used in anæmic cases, but has to be given with caution owing to its tendency to set up constipation or gastric irritation. It is generally prescribed as follows: Tinct. ferri perchlor. ♏xv., glycerine ʒi., spirit chlorof. ♏x., aq. ad ℥i., to be taken three times a day after meals. Mag. sulph. ʒ ss. may be added to prevent its constipating effect. Whichever of these drugs be prescribed, the condition of the stomach must never be lost sight of, and although the above indications are useful in guiding the course of treatment, many cases do equally well, or even better, when depending only on good food and cod liver oil.

*Cough.*—As long as the diseased lung continues to secrete cough is inevitable, and it is useless to attempt to check it by other than constitutional means. The frequent use of opiates has a great tendency to destroy the appetite and set up constipation, so they should be given as sparingly as possible. It is, however, legitimate to give special drugs for the relief of an irritable or unnecessary cough, or one which prevents the patient from getting the necessary amount of sleep. For checking an irritable cough, it is often sufficient to add tinct. camph. co., ʒ ss., to the medicine; and the following pill, prescribed by the late Dr. Milner Fothergill, is also extremely useful for this purpose: ℞. pulv. guaiaci, gr. i.; pulv. ipecac., pulv. opii, aa. gr. ss.; pil. scillæ co., gr. ii.; fiat pil. to be given when the cough is troublesome. For relieving cough at night, a morphia linctus, containing liq. morph. hyd. ♏iii.; spir. chlorof., ♏iii.; glycerine, aquæ a.a. ʒ ss., is given two or three times if necessary; or the following draught at bedtime: ℞. liq morph. hyd. ♏xii.; pot. iod. gr. iii.; glycerine, ʒi.; aq chlorof. ℥i. In other cases more benefit is derived from a few drops of chlorodyne or laudanum. Another useful drug, which may be used when the above measures have failed, is codeine, in $\frac{1}{2}$ to 2 gr. doses, as it is less likely to be followed by depression or constipation than morphia. In very advanced cases, where treatment can only be palliative, less hesitation is required in giving opiates or anything else that will give the patient any relief.

*Dyspnœa.*—Shortness of breath on exertion is a frequent symptom, but except in advanced stages is generally relieved by rest, and avoidance of other than gentle exercise. When troublesome, independently of exertion, the patient should be kept entirely in bed, propped up in a sitting position by extra pillows, with hot-water bottles to the feet if necessary. Stimulants are of great service in this condition, and are best given in the form of brandy. Occasional doses of the following mixture often give great relief. ℞. tinct. nux. vom. ♏x., spir. chlorof. ♏xv., amm. carb. gr. v. infus. caryophylli. ℥i. In very advanced cases where dyspnœa is the most distressing symptom, temporary relief may be obtained by the following—Spir. amm. aromat., spir. ætheris. aa. ʒss. aq. camph. ℥i. in frequent doses, alternating with about an ounce of brandy. Ether or brandy are sometimes injected subcutaneously as a last resource.

*Pain.*—Phthisical patients are subject to numerous aches and pains, of which it is sometimes difficult to determine the cause. If they are situated in the region described under dyspepsia they are probably gastric in origin and must be treated accordingly; if over an area of the chest where physical signs can be made out, and especially if increased on coughing or deep breathing, they are probably pleuritic. Pain due to pleurisy is very amenable to local treatment, being generally relieved by the application of liniment or tincture of iodine, or a small blister is even more efficacious, over the part complained of for two or three nights in succession. Strapping the affected side of the chest is a good remedy when counter-irritants are useless, but if local treatment fails altogether ten grains of Dover's powder or some other form of opiate may be given. Pain may also be due to the supervention of pneumothorax (the general treatment of which will not be discussed), and is usually treated here with morphia, either by the mouth or hypodermically.

*Night Sweats.*—A moderate amount of sweating may be checked by the use of creasote, hypophosphite of lime, or the mineral acids as mentioned above. The patient should not be overloaded with bedclothes, and care must be taken to avoid draughts. In more severe cases the body may be sponged with tepid water or vinegar at bedtime, and again later on in the night if necessary. A pill containing sulphate of atropine gr. $\frac{1}{100}$ to $\frac{1}{50}$ (beginning with the smaller quantity), taken at bedtime is generally sufficient to check the sweating, or atropine combined with morphia as in the following pill, another legacy of the late Dr. Fothergill: Atrop. sulph., gr. $\frac{1}{60}$; morph. hyd., gr. $\frac{1}{4}$; pulv. capsici, gr. i.; pil aloes and myrrhæ, gr. iii. Should atropine cause any dryness of the throat or dilatation of the pupil or fail to check the sweating, a trial is generally given to oxide of zinc, four grains in a pill made up with confection of roses and taken every night. Agaricin, grain $\frac{1}{12}$ to $\frac{1}{6}$ has also been used with success when the other measures have failed, either by itself or combined with Dover's powder. Its reputed laxative effect has not been observed here.

*Diarrhœa.*—In the later stages of phthisis diarrhœa is generally the result of ulceration or lardaceous degeneration of the bowel, and often most intractable, the patient sinking rapidly under its exhausting influence. The stools often contain particles of undigested food, which points to regulation of the diet as the first consideration in treatment. Milk and lime water, arrowroot and thickened broths, to which three or four grains of pepsine or some other peptonizing agent may be added, constitute the best diet. Brandy is given in proportion to the age and feebleness of the patient, large quantities being often necessary. A flannel binder round the abdomen is of service, and hot fomentations may be applied to relieve pain. A slight tendency to diarrhœa will sometimes yield to treatment under the hypophosphites of calcium, cinchona bark, and mineral acids, but when severe, opium, either alone or combined with some astringent is the only drug which can be relied on, and often enough this also fails to give more than temporary relief. A mixture containing tinct. opii. ♏x. (or more), tinct. catechu ʒi., decoct. hæmatoxylin ℥i., is generally given here, either every four hours or after each loose motion. A pill containing argenti oxidi, pulv. opii aa. gr. ss., mucilag. q.s., every four hours is sometimes successful, and there are few preparations of opium which have not been used in their turn. Hypodermic injections of morphia are given as a last resource.

*Hæmoptysis.*—During the actual attack there is not time to do much, but the position of the patient is of some importance. Death occurring during an attack of hæmoptysis is almost always due to impaction of blood clots in the trachea or main bronchi and consequent asphyxia, and therefore if the patient show any symptoms of suffocation he should be placed across the bed with the head hanging over the edge, and slapped several times on the back until the clot is dislodged. Generally, however, the hæmoptysis ceases gradually, and the chief aim of treatment is to prevent a recurrence. The patient is kept absolutely at rest in bed, and placed on a diet consisting only of milk, soda water, and ice; a hypodermic injection of morphia (gr. 1-6 to 1-3) is generally given at once, the chief object being to depress the action of the heart, and to check cough. Opium and purgatives are the drugs chiefly relied on, and are frequently prescribed as follows: Tinct. opii. ♏x., mag. sulph. ʒi., inf. rosæ. acid ℥i., to be taken every four hours, and in some cases, especially with a pulse of high tension, two or three grains of calomel are of service. If, in spite of this treatment, there is persistent recurrence of hæmoptysis, hypodermic injections of ergotine (gr. ii.), or the addition to the above medicine of liquid extract of ergot (♏xx) may be tried, and the frequent inhalation of turpentine on a piece of lint has sometimes appeared beneficial, and at any rate can do no harm. The application of an ice bag to the chest over the lung from which the hæmorrhage is supposed to occur has been frequently employed here, but without any obvious benefit. Rest in bed and milk diet should be continued until all traces of blood in the sputum have ceased, when gradual relaxation in these respects may be allowed, but the reappearance of even a tinge of blood should be regarded as a danger signal. Some patients expectorate blood-stained sputum for days or weeks without having any worse attack, but the same caution should be exercised with regard to them as in severer cases. After repeated attacks of hæmoptysis there may be so much prostration that stimulants become absolutely necessary, and in other cases it is often difficult to decide whether it is consistent with safety to withhold them—the condition of the heart, as indicated by the pulse and character of the first sound, is the best guide on this point.

